# The mental health and psychological wellbeing of South African athletes and coaches during the COVID-19 pandemic: lessons learned from their health experience

**DOI:** 10.17159/2078-516X/2025/v37i1a16401

**Published:** 2025-06-15

**Authors:** RL van Niekerk, K Tsebe

**Affiliations:** 1 Department of Psychology, University of Fort Hare, South Africa; 2 Department of Psychology, University of South Africa, South Africa

**Keywords:** psychological distress, lockdown, social, emotional

## Abstract

**Background:**

During the COVID-19 pandemic lockdown, athletes’ and coaches’ participation in sport was restricted severely.

**Objectives:**

This study explore the mental health and wellbeing of athletes and coaches in South Africa during the COVID-19 pandemic at the end of adjusted level 3 lockdown in February 2021. At the time, participants were restricted from engaging in sports activities for a year.

**Methods:**

A convenience sample of 135 athletes (mean age= 36.2±16.8) and 110 coaches (mean age=44.0±14.0) from 37 sporting codes participated in the study. They were invited via their federations to complete the Kessler Psychological Distress Scale and Mental Health Continuum – Short Form. Descriptive results, correlations and group differences were determined. A direct logistic regression was done to predict psychological distress for athletes and coaches.

**Results:**

Psychological distress was observed for athletes (46%) and coaches (47%), while 45% of athletes and 46% of coaches were flourishing under conditions of the pandemic. The social wellbeing of more athletes’ (25%) and coaches’ (28%) was disrupted than their emotional and psychological wellbeing during lockdown. The mental wellbeing of athletes (OR=0.86; CI 95%=0.81–0.91: p=0.0001) and coaches (OR=0.93; CI 95%=0.89–0.97: p=0.0001) seemed to mitigate their psychological distress, while female athletes (OR=4.97; CI 95%=1.62–15.18: p=0.005) were almost five times more likely to experience psychological distress than male athletes.

**Conclusion:**

Almost half of the participating athletes and coaches showed high psychological distress, while high mental wellbeing seemed to protect athletes and coaches from negative mental health outcomes during the pandemic. Female athletes and coaches were identified as high-risk groups for mental health challenges during lockdown conditions.

The Severe Acute Respiratory Syndrome Coronavirus 2 (SARS-CoV-2), responsible for the COVID-19 pandemic, spanning from 26 March 2020 to 4 April 2022, disrupted the daily lives of physical activity, mental health, and overall wellbeing of individuals worldwide.^[1]^ In South Africa, the pandemic resulted in the implementation of restrictive lockdown measures that included a ban on sporting activities, with a gradual resumption of training and sports participation as lockdown restrictions changed.^[2]^ The government implemented a system of five distinct lockdown levels, each characterised by varying degrees of restriction on sports participation for athletes and coaches. The rollout of the lockdown started with Level 5 on March 26, 2020, which entailed an immediate and comprehensive prohibition on public sporting and exercise activities. Subsequently, on May 1, 2020, the government transitioned to Level 4 lockdown, permitting limited public exercise opportunities under stringent public health guidelines. Level 3, which started on June 1, 2020, marked the reinstatement of non-contact sports activities, albeit under rigorous adherence to public health protocols. In contrast, Level 2, implemented on August 18, 2020, permitted small-group training sessions while still enforcing strict public health measures, with the additional stipulation that no spectators were allowed to attend. As of September 21, 2020, with the initiation of Level 1, a gradual return to unrestricted sports activities was facilitated, albeit with certain limitations placed on spectator attendance.^[2]^ However, the government subsequently imposed an adjusted Level 3 lockdown, effective from December 29, 2020, to February 28, 2021, followed by a series of adjustments across Levels 4 to 1 throughout the remainder of the year. It was only on April 4, 2022, that the lockdown measures were lifted, allowing coaches and athletes to begin normalising their sports participation.^[2]^

These lockdown measures were implemented to safeguard society as a whole and mitigate the spread of infections. Consequently, the lockdown necessitated the cancellation of all sporting events, training sessions, leagues, and related activities, thereby impacting coaches and athletes across a wide spectrum of sports disciplines.^[3]^ The consequences of the pandemic should not be underestimated,^[1]^ particularly considering the presence of poverty, gender, and structural inequality, unemployment, limited access to mental health services, and an increasing disease burden in South Africa, which did exacerbate the situation.^[4]^

Mental health encompasses an individual’s state of wellbeing, including their ability to recognise their capabilities, cope with normal life stresses, work productively, contribute to their community, and experience emotional, social, and psychological wellbeing.^[5]^ Mental wellbeing reflects the flourishing or languishing state of an individual’s emotional, social, and psychological dimensions.^[6]^ The two-continua model of mental health used in this study integrates both the perspective of mental illness (mental disorders) and mental health (wellness) to understand overall mental wellbeing.^[7]^ The importance of addressing mental health in athletes is underscored in various consensus statements, such as those from the South African Sports Medicine Association (SASMA).^[3]^ However, similar attention is not often given to coaches, leading to their mental health being frequently overlooked.

Concerns were raised regarding the impact of the COVID-19 pandemic on the mental health of elite athletes, attributable to various factors such as diminished physical activity, isolation from teammates, detachment from the athletic community, limited interactions with coaches, and a lack of social support.^[4]^ An examination of the extant literature,^[8,9]^ focusing on the mental wellbeing of elite athletes primarily in European, American, Asian, and Australian countries (with one South African study included), has determined that the pandemic has had a detrimental impact on the mental health and overall wellbeing of athletes. This impact has been observed to manifest across a spectrum, ranging from mild to equally severe when compared to the general population. In addition, elevations in levels of depression, anxiety, and stress among athletes, with a heightened vulnerability were observed among female and elite athletes in particular.^[9]^ The prevalence rates for anxiety and depression within the elite athlete population have been exhibited in a wide range, spanning from 21% to 48% and 17% to 57%, respectively.^[10]^ Notably, approximately half of all elite athletes encountered some form of mental health challenge,^[11]^ regardless of potential mitigating factors such as physical activity, mental resilience, and adaptive coping skills.^[12]^

A South African study on elite and semi-elite athletes during the pandemic indicated that most athletes trained in isolation (61%) and reported changes in sleeping patterns (79%), depression (52%), and lack of motivation (55%).^[13]^ Research on cricket players demonstrated moderate levels of anxiety, with some individuals experiencing elevated levels of anxiety, depression, and stress.^[14]^ The introduction of a biologically safe environment among football players resulted in significant reductions in anxiety and depression, with mental toughness and resilience playing crucial roles in their adaptation to the circumstances.^[15]^ However, studies focusing on the impact of the pandemic on the mental health and wellbeing of sports coaches have been largely neglected. A study conducted in Italy reported increased levels of perceived stress among both male and female coaches during the pandemic, with male and elite coaches exhibiting better coping skills compared to their female and non-elite counterparts.^[16]^

The COVID-19 pandemic had direct and indirect psychological and social consequences for the mental health of athletes and coaches, which have yet to be thoroughly explored, particularly among athletes and coaches in South Africa. Therefore, there is a need to investigate and report on the mental health and wellbeing of South African athletes and coaches during the pandemic. This study aims to assess and compare the mental health and wellbeing of athletes and coaches during the COVID-19 pandemic and reflect on the lessons learned from this experience.

## Methods

### Study design

A study on the mental health and wellbeing of South African athletes and coaches was conducted during the adjusted lockdown level 3 in South Africa from December 2020 to February 2021. All sporting events had been restricted for 12 months since lockdown started in March 2020. Invitations to recruit participants were extended to the management of all sports federations in South Africa, which are listed on the South African Sports Confederation and Olympic Committee (SASCOC) website. Federations that decided to participate were provided with a link to an online research platform and requested to make it available to their members. The study included only athletes and coaches who were 18 or older and provided their consent.

### Sample

A convenient sample of 245 participants responded to invitations sent through their respective Sport Federations, indicating their willingness to participate in the study. This group comprised 135 athletes (55%) and 110 coaches (45%) from 37 different sporting codes who participated in the study after receiving the invitations.

### Research instruments

Participants were asked to complete an online questionnaire created by the authors using SurveyMonkey (http://www.surveymonkey.com). Before accessing the questionnaire, participants were required to provide informed consent electronically. The biographical section of the questionnaire collected information on variables such as gender, age, sporting code, level of sports participation, home environment, and lockdown conditions. The section on mental health and wellbeing included the following tests:

- The Kessler Psychological Distress Scales (K-10):^[17]^ This 10-item test assessed the psychological distress experienced by athletes and coaches. The K-10 measures non-specific psychological distress and screens for mental health concerns, particularly depression and anxiety. Participants responded on a 5-point Likert scale ranging from 1 (“none of the time”) to 5 (“all of the time”).^[17]^ A total psychological distress score was calculated for each participant that was subjected to analysis in this study. The K-10 is widely used as a screening tool for mental health in various populations and has demonstrated excellent reliability and validity (Alpha=0.907) in a South African university population during the COVID-19 pandemic.^[18]^
- Mental Health Continuum – Short Form (MHC-SF):^[6]^ This 14-item questionnaire was used to evaluate the wellbeing of athletes and coaches. The instrument provides measures for three sub-scale scores, namely psychological-, emotional-, and social wellbeing, using a 6-point Likert scale, as well as an overall wellbeing score. The overall wellbeing score is determined by summing the three subscale scores to provide a general measure of participants’ overall wellbeing. The MHC-SF has been validated in South Africa^[19]^ and has shown acceptable to high reliability across all sub-scales. During the COVID-19 pandemic, the instrument demonstrated high reliability (Alpha=0.929) in a South African university population.^[18]^

### Statistical analysis

Data analysis was conducted using SPSS version 25^[20]^ statistical package. The mental health (items 1–10 of the K10) and wellbeing (items 1–14 of the MHC-SF) of the athletes and coaches were reported through descriptive statistics, including frequencies, percentages, means, and standard deviations. The results of the scales and sub-scales of the psychometric tests for mental health (K10) and wellbeing (MHC-SF) were calculated according to the scoring norms based on their respective test manuals. Relationships between mental health, wellbeing and age were determined using Pearson product-moment correlations. Group comparisons were performed using independent samples t-tests to compare the mental health and wellbeing outcomes of coaches and athletes and gender groups. The internal consistency of the scales was evaluated using Cronbach’s Alpha. Additionally, a binary logistic regression analysis was conducted to identify factors that predict mental health concerns among athletes and coaches separately to allow for comparative discussion of the two groups. Mental health concerns were dichotomised using the results of the Kessler Psychological Distress Scales (K-10)^[17]^ to classify participants either with (“yes”) or without (“no”) psychological distress. Psychological distress was the predictor variable. Age, gender, mental wellbeing, having enough space to train, having company during lockdown and being elite athletes/coaches, were employed as outcome variables. A significance level of 0.05 at a 95% confidence interval was used.

## Results

### Biographical traits and sport context of the participants

The results of 245 athletes (mean age=36.2±16.8 years) and coaches (mean age=44.0±14.1 years) from 37 sporting codes were analysed and presented in Table 1, indicating a diverse distribution of participants according to gender, language, geographical factors (province and location) and sport participation (type of sport, competitive level, and participation level). The sporting codes represented were tennis (23%), netball (15%), lifesaving (6.1%), long distance running (5.7%), swimming (4.5%), canoeing (4.5%), softball (4.1%), ice hockey (3.7%), table tennis (2.9%), triathlon (2.9%), figure skating (2.9%), soccer (2.4%), track and field athletes (2.4%), kayaking (2.4%), jukskei (2.4%), cycling (2.0%), karate/martial arts (2%), golf (0.8%), baseball (0.8%), power walking (0.8%), canoe marathon (0.8%), field hockey (0.8%), cricket (0.4%), yoga (0.4%), rugby (0.4%), squash (0.4%), pétanque (0.4%), bass angling (0.4%), canoe polo (0.4%), surfing (0.4%), rowing (0.4%), aquatics (0.4%), biathlon (0.4%), taekwondo (0.4%), sailing (0.4%), paddling (0.4%), and weight-lifting (0.4%).

### Environmental conditions during lockdown

Environmental information gathered during the COVID-19 pandemic lockdowns is summarised in Table 2. Participants were asked to indicate whether they found their home environments to be conducive for training during lockdown. Most athletes (92%) and coaches (86%) answered affirmatively. Findings reveal that a minority of athletes (5.2%) and coaches (10%) reported being in solitary conditions during the lockdown period, while most athletes (82%) and coaches (86%) resided in home environments, such as houses with yards. Athletes engaged in training activities for an average of 10.2±7.3 hours per week, whereas coaches dedicated approximately 16.0±10.4 hours per week to training. The quality of training conditions gradually improved as restrictions were lifted. The percentage of athletes and coaches reporting satisfactory training conditions increased from 30% and 31% at lockdown level 5 to 80% and 83% at lockdown level 1, respectively.

### Mental health and wellbeing

High reliability was obtained for both the Kessler Psychological Distress Scale^[17]^ (Cronbach Alpha=0.921) and the Mental Health Continuum – Short Form (MHC-SF)^[6]^ (Cronbach Alpha=0.918). No statistically significant differences (p>0.05) were found between athletes and coaches for mental health and wellbeing measures on all scales and sub-scales (see Table 3).

Mental health and wellbeing categories are presented in Table 4, providing insights into the participants’ psychological experiences. Psychological distress is a measure that captures the extent of distress aligned with a diagnosis of depression and/or anxiety disorder. It was found that a considerable proportion of athletes (46%) and coaches (47%) experienced mild to severe levels of psychological distress. In terms of mental wellbeing, which gauges an individual’s psychological functioning and overall sense of thriving or struggling, a similar proportion of athletes (44%) and coaches (46%) flourished during the pandemic. Conversely, a small subset of athletes (10%) and coaches (8.2%) were identified as languishing. Regarding specific dimensions of wellbeing, less than 10% of coaches and athletes reported low emotional and psychological wellbeing, while a higher proportion of athletes (25%) and coaches (28%) indicated low social wellbeing. Notably, the impact of the pandemic appeared to have a greater influence on the social wellbeing of athletes and coaches compared to their emotional and psychological wellbeing.

### Group differences and the relationship between mental health and wellbeing

Statistically significant differences were found between gender groups (Table 5) in terms of psychological distress, with male participants (18.8±7.6 arbitrary units (AU) exhibiting lower distress levels compared to female participants (21.3± 8.1 AU, p=0.013). Furthermore, participants who reported having satisfying training space, reported significantly higher overall-(p=0.001), emotional-(p=0.007) social-(p=0.017) and psychological wellbeing (p=0.001) than those who reported not having satisfying training space. When asked whether their living space during the pandemic was conducive, those who indicated “yes” reported significantly higher overall-(p=0.020), emotional-(p=0.012) and social wellbeing (p=0.014) than those who said “no”. Athletes and coaches who reported that they were alone during isolation, reported significantly lower psychological wellbeing (p=0.036) than those who had companionship during isolation.

A strong negative correlation was observed between psychological distress and overall wellbeing (r=−0.624; p=0.0001) and age (r=−0.150, p=0.019) in Table 6. The results indicated that in participants who were older or had higher observed. Both wellbeing and age (maturity) seems to mitigate psychological distress.

### Factors predicting the psychological distress of athletes and coaches

A logistic regression was conducted for athletes and coaches to determine the impact of several factors on the likelihood that they might experience psychological distress during the COVID-19 pandemic (Table 7). The proposed model included six independent variables, namely overall mental wellbeing (as a measure of participants’ emotional-, social-and psychological wellbeing), having sufficient training space, gender, having company during lockdown, status (elite athlete or coach) and age.

The full model for athletes including all six predictors was statistically significant, χ^2^ (6, N=133)=79.20, p<0.0001, indicating that the model could distinguish between athletes who experienced or did not experience psychological distress. The model explained between 42% (Cox and Snell R square) and 55% (Nagelkerke R squared) of the variance for psychological distress and correctly classified 81% of athletes. It was observed in Table 7, that only two of the independent variables, namely mental wellbeing and gender made a statistically significant contribution to the model. The strongest predictor of psychological distress among athletes was gender, recording an odds ratio of 4.97. This implied that female athletes were over four times more likely to report psychological distress during the pandemic than their male counterparts. The odds ratio indicated that with every point that mental wellbeing increased, the athletes’ odds for psychological distress decreased with 0.86, as psychological distress was chosen as the predictive variable.

Similarly, the full model for coaches including all six predictors was statistically significant, χ^2^ (6, N=108)=25.86, *p*<0.000, indicating that the model was able to distinguish between coaches who experienced or did not experience psychological distress during the pandemic. The model explained between 21% (Cox and Snell R square) and 28% (Nagelkerke R squared) of the variance for psychological distress among coaches and classified 72% of coaches correctly. It was observed in Table 4, that only two of the independent variables, namely mental wellbeing, and age made a statistically significant contribution to the model.

The strongest predictor of psychological distress among coaches was age, recording an odds ratio of 0.96. This implied that for every one-year increase in a coach’s age, they are likely to experience an increase of 0.96 in their psychological distress scores during the pandemic. The odds ratio indicates that for every point increase in mental wellbeing, the coaches’ odds for psychological distress decrease by 0.93, as psychological distress was chosen as the predictive variable.

## Discussion

Regardless of the prevailing pandemic conditions, most of the participating athletes and coaches in this study reported living in a house with a yard that was conducive for training during lockdown and experienced an expansion of training space as the lockdown restrictions gradually eased. They also highlighted the importance of having companionship during the pandemic. A small fraction of athletes and coaches reported being alone during the lockdown periods. The limitations imposed on athletes due to isolation and their living environments had a significant impact on their mental health during the pandemic, leading to various mental health challenges. ^[4, 8, 9, 13]^

In terms of psychological distress, approximately half of the sample, both athletes (46%) and coaches (47%), reported mild to severe levels of distress as a screening measure for depression and anxiety. Conversely, a higher proportion of athletes (45%) and coaches (46%) demonstrated positive mental wellbeing, indicating flourishing. Although no direct comparison was made, these findings were higher than the depression and anxiety rates observed in other studies prior to the COVID-19 pandemic^[21]^ and fairly similar to results from other studies conducted during the pandemic.^[9, 10]^ The data in this study may indicate that psychological distress rates are high relative to those published in other studies among South African cricket players^[14]^ and athletes.^[13]^

The social wellbeing of athletes and coaches was much lower than their psychological- and emotional wellbeing during the pandemic, suggesting that their social wellbeing was much more affected than their emotional and psychological wellbeing at the time. This disparity can be attributed to the enforced isolation, social distancing measures, and limited access to teammates during the lockdown periods.^[22]^ Moreover, female athletes and coaches exhibited significantly higher levels of psychological distress compared to their male counterparts, suggesting a greater vulnerability to mental health problems among women during the COVID-19 pandemic.^[23]^ The presence of satisfying environments had a significant positive impact on wellbeing scores, indicating that improved living conditions during periods of isolation contributed to better mental health outcomes.^[22]^

Mental wellbeing was a significant predictor of psychological distress for both athletes and coaches. Lower levels of mental wellbeing were associated with increased psychological distress, indicating mitigation in the development of anxiety and depression. ^[12,13]^ Among coaches, age played a significant role in predicting psychological distress, whereas gender was a significant predictor among athletes. Female athletes were more than four times more likely to experience elevated levels of psychological distress during the pandemic compared to their male counterparts, as confirmed in other research.^[23]^ Similar results on the mental health of athletes and coaches, primarily stemming from disruptions to their personal lives, training regimens, career prospects, and income losses^[9]^, were reported.^[3, 13, 15]^ This situation may have been exacerbated by the insufficient availability of mental health resources in Africa^[4]^, with the expectation of prolonged psychological distress even after the conclusion of the pandemic. Consequently, there is a pressing need for sustainable psychological support for both athletes and coaches to mitigate mental health outcomes on the sports community during a pandemic. Limitations of the study included participant bias, gatekeeper control by federations who opted not to participate in the study and unavailability of mental health and well-being of athletes and coaches prior to the pandemic.

## Conclusion

The mental health and wellbeing of athletes and coaches during the COVID-19 pandemic showed notable challenges for their social wellbeing and psychological distress, with some lessons learned about their wellbeing during times of isolation. The presence of mitigating factors such as satisfying living conditions and robust mental wellbeing could potentially offer protection and support. It is crucial to recognize that female athletes and individuals with lower levels of mental wellbeing are particularly vulnerable and may require additional mental health care and support. Sports federations need to be cognizant of the mental health challenges faced by athletes and coaches, and it is imperative to implement mental health care programs to assist athletes and coaches during a pandemic. Furthermore, the mental health and wellbeing of coaches should not be overlooked, as they exhibited similar levels of vulnerability compared to that of athletes during the COVID-19 pandemic.

## Figures and Tables

**Table 1:** Biographical and sporting characteristics of athletes and coaches in the sample

Variable	Group	Athletes		Coaches	

		n	%	n	%

Gender	Male	68	50	57	52
	Female	67	50	52	48

Language	English	61	46	53	49
	Afrikaans	47	36	40	37
	Sepedi	10	7.6	3	2.8
	isiZulu	5	3.8	3	2.8
	isiXhosa	3	2.3	2	1.8
	Tshivenda	2	1.5	1	0.9
	Setswana	1	0.8	4	3.7
	Other	3	2.8	3	2.7

Province	Gauteng	34	25	42	39
	Western Cape	31	23	28	26
	KwaZulu-Natal	29	22	10	9.2
	Northwest	17	13	6	5.5
	Limpopo	9	6.7	4	3.7
	Eastern Cape	8	5.9	10	9.2
	Mpumalanga	4	3.0	4	3.7
	Free State	2	1.5	2	1.8
	Northern Cape	1	0.7	3	2.8

Location	Urban	107	79	97	88
	Rural	28	21	13	12

Sport type	Individual sport	85	63	84	77
	Team sport	49	37	25	23

Competative level	Club/school level	48	36	39	35.5
	Professional Team	3	2.2	1	0.9
	Sport Academy	1	0.7	11	10
	High Performance Team	5	3.7	4	3.6
	Provincial	25	19	17	16
	National	17	13	26	24
	International	35	26	12	11

Participation	Amateur level	89	66	66	61
	Professional level	45	34	43	39

Age		**Mean±SD**		**Mean±SD**	

		36.2±16.8		44.0±14.1	

SD, standard deviation

**Table 2:** Environmental conditions during COVID-19 lockdown

Conditions	Group	Athletes		Coaches	

		n	%	n	%

Condusive home environment	Yes	123	92	95	86
	No	11	8.2	15	14

Home conditions	A house with a yard	110	82	95	86
	A house with no yard	6	4.5	-	-
	A flat	6	4.5	11	10
	A one room apartment	4	3.0	4	3.6
	An informal settlement	1	0.7	-	-
	Farm	7	5.2	-	-

Satisfying training space	Lockdown 5	40	30	34	31
	Lockdown 4	46	35	35	34
	Lockdown 3	75	56	69	66
	Lockdown 2	96	73	78	74
	Lockdown 1	106	80	86	83
	Adjusted Lockdown 3	70	60	58	62

Having company	Yes	128	95	99	90
	No	7	5.2	11	10

**Table 3:** Group comparisons on mental health and wellbeing for athletes and coaches

Variable	Group	n	Mean±SD	p-value

Psychological distress	Athlete	135	20.3±8.4	0.581
	Coach	110	19.8±7.5	

Overall wellbeing	Athlete	135	45.2±13.6	0.767
	Coach	110	44.7±13.4	

Emotional wellbeing	Athlete	135	10.9±3.2	0.373
	Coach	110	10.5±3.0	

Social wellbeing	Athlete	135	12.6±6.0	0.796
	Coach	110	12.4±5.7	

Psychological wellbeing	Athlete	135	21.8±6.2	0.963
	Coach	110	21.8±6.2	

**Table 4:** Frequencies of mental health and wellbeing categories

Scale	Group	Athletes		Coaches	

		n	%	n	%

Psychological distress	Normal	73	54	58	53
	Mild Psychological distress	19	14	22	20
	Moderate Psychological distress	19	14	21	19
	Severe Psychological distress	24	18	9	8.2

Overall wellbeing	Languishing	14	10	9	8.2
	Moderate	60	44	50	46
	Flourishing	61	45	51	46

Emotional welbeing	Low	11	8.1	7	6.4
	Moderate	39	29	43	39
	High	85	63	60	55

Social welbeing	Low	34	25	31	28
	Moderate	69	51	52	47
	High	32	24	27	25

Having company	Low	7	5.2	8	7.3
	Moderate	41	30	31	28
	High	87	64	71	65

**Table 5:** Group differences for mental health and wellbeing

Mental health (AU)	Gender			Satisfying training space			Condusive living space			Having companionship		

	Group	Mean±SD	p-value	Group	Mean±SD	p-value	Group	Mean±SD	p-value	Group	Mean±SD	p-value

Psychological distress	Male	18.8±7.6	0.013*	Yes	19.6±8.4	0.253	Yes	19.9±8.1	0.509	Yes	19.8±7.9	0.056
	Female	21.3±8.1		No	20.9±7.4		No	21.0±7.3		No	23.6±8.9	

Overall wellbeing	Male	46.2±12.9	0.206	Yes	47.2±13.6	0.001*	Yes	45.7±13.2	0.020*	Yes	45.4±13.0	0.100
	Female	44.0±13.9		No	41.4±12.7		No	39.2±14.8		No	40.0±17.7	

Emotional wellbeing	Male	10.9±3.1	0.242	Yes	11.1±3.2	0.007*	Yes	10.9±2.9	0.012*	Yes	10.8±3.0	0.070*
	Female	10.5±3.1		No	10.0±2.9		No	9.3±3.7		No	9.4±3.7	

Social wellbeing	Male	12.9±5.8	0.286	Yes	13.2±6.2	0.017*	Yes	12.9±5.9	0.014*	Yes	12.6±5.8	0.542
	Female	12.2±5.9		No	11.4±5.2		No	9.9±5.1		No	11.7±7.2	

Psychological welbeing	Male	22.3±5.9	0.251	Yes	22.8±5.9	0.001*	Yes	21.9±6.1	0.137	Yes	22.0±5.9	0.036
	Female	21.3±6.4		No	20.0±6.3		No	20.1±7.1		No	18.8±8.5	

* Statistical significance, p<0.05

**Table 6:** Group comparisons on mental health and wellbeing for athletes and coaches

Variable		Psychological distress	Overall wellbeing	Age

**Psychological distress**	Correlation	1		
	Sig.			

**Overall wellbeing**	Correlation	−0.62**	1	
	Sig.	0.0001		

**Age**	Correlation	−0.15*	0.2	1
	Sig.	0.019	0.775	

* Statistical significance, p<0.05

** Statistical significance, p<0.01

**Table 7:** Logistic regression predicting the likelihood of psychological distress for athletes and coaches

Group	Variable	B	SE	Wald	df	p-value	Odds ratio	95% CI for EXP(B)	

								Lower	Upper

**Athletes**	Mental wellbeing	−0.15	0.03	30.10	1	0.000*	0.86	0.81	0.91
	Training space	−0.33	0.50	0.43	1	0.513	0.72	0.27	1.91
	Gender	1.60	0.57	7.93	1	0.005*	4.97	1.63	15.18
	Company	−0.58	1.16	0.25	1	0.618	0.56	0.06	5.43
	Elite	0.62	0.49	1.61	1	0.205	1.87	0.71	4.89
	Age	0.02	0.02	1.72	1	0.190	1.02	0.99	1.06
	Constant	5.69	1.76	10.51	1	0.001*	296.99		

**Coaches**	Mental wellbeing	−0.08	0.02	12.69	1	0.000*	0.93	0.89	0.97
	Training space	0.32	0.49	0.42	1	0.518	1.37	0.53	3.58
	Gender	0.16	0.44	0.14	1	0.712	1.18	0.49	2.81
	Company	−0.66	0.77	0.74	1	0.391	0.52	0.11	2.34
	Elite	0.04	0.45	0.01	1	0.935	1.04	0.43	2.53
	Age	−0.04	0.02	5.20	1	0.023*	0.96	0.93	1.00
	Constant	−0.08	0.02	12.69	1	0.000*	0.93	0.89	0.97

* Statistical significance, p<0.05.

Variable(s) entered on step 1: Mental wellbeing, Training Space, Gender, Company, Elite, Age.

B, Beta Coefficient; SE, Standard Error; Wald, Wald Chi-Square Statistic; df, Degrees of Freedom
